# Interstitial 6q21q23 duplication - variant of variable phenotype and incomplete penetrance or benign duplication?

**DOI:** 10.1186/s13039-016-0253-9

**Published:** 2016-06-02

**Authors:** Malgorzata I. Srebniak, Laura J. C. M. van Zutven, Florence Petit, Sonia Bouquillon, Ilse P. J. van Heel, Maarten F. C. M. Knapen, Jerome M. J. Cornette, Andreas Kremer, Diane Van Opstal, Karin E. M. Diderich

**Affiliations:** Department of Clinical Genetics, Erasmus MC, Ee2475, Wytemaweg 80, 3015 CN Rotterdam, The Netherlands; Department of Clinical Genetics, University Hospital, Lille, France; Department of Cytogenetics, University Hospital, Lille, France; Department of Obstetrics and Gynecology, subdivision Obstetrics and Prenatal Medicine, Erasmus MC, Rotterdam, The Netherlands; Stichting Prenatale Screening Zuidwest Nederland, Wytemaweg 80, Na-1509, 3015, GE Na-1503 Rotterdam, The Netherlands; Department of Bioinformatics Erasmus MC, Rotterdam, The Netherlands

**Keywords:** Interstitial 6q22.1q23.2 duplication, Interstitial duplication 6q21q22.33, 16p11.2 microduplication, Susceptibility locus, Second hit hypothesis

## Abstract

**Background:**

Chromosome 6q duplication syndrome is a chromosome abnormality associated with characteristic phenotypic features such as intellectual disability (ID), short stature, feeding difficulties, microcephaly, dysmorphic features (prominent forehead, downslanting palpebral fissures, flat nasal bridge, tented upper lip, micrognathia, short webbed neck) and joint contractures. Only a few cases of pure partial 6q trisomy have been published and the severity of the phenotype seems to depend on the breakpoint position. Unfortunately, most of these cases were identified using karyotyping or FISH, so breakpoints at the molecular level and thus gene content are not known.

**Cases presentation:**

We report the first two families with an interstitial 6q duplication identified by karyotyping where the gene content and breakpoints were characterized with microarray. In family 1, the 6q22.1q23.2 duplication was detected in a female patient with ID. In family 2, the 6q21q22.33 duplication was identified in a male fetus with multiple congenital malformations. In both families, the duplication seems to show phenotypic heterogeneity and in family 1 also incomplete penetrance suggesting the co-existence of an “additional hit” in affected patients. This “additional hit” was identified in the first family to be a microduplication in 16p11.2, a known susceptibility locus (SL) for neurodevelopmental disorders, that co-segregated with an abnormal phenotype in the affected family members.

**Conclusions:**

Our study shows that interstitial 6q21q23 duplication may represent a private variant that is benign, but may also contribute to developmental disorders of variable expressivity in a “multi-hit” model. Finding the “additional hit” within the family is therefore very important for genetic counseling and assessment of the CNV penetrance within the particular family.

## Background

The chromosome 6q duplication syndrome is a known chromosome abnormality associated with characteristic phenotypic features such as severe intellectual disability (ID), short stature, feeding difficulties, microcephaly, dysmorphic features (prominent forehead, downslanting palpebral fissures, flat nasal bridge, tented upper lip, micrognathia, short webbed neck) and joint contractures [1–5]. Most reported patients carry unbalanced translocations leading to a partial trisomy 6q and a partial monosomy of another chromosome. In these cases the monosomic region of another chromosome is also of great influence on the patient’s phenotype. There are only several cases of pure partial 6q trisomy and the severity of the phenotype seems to depend on the breakpoint position. In general, terminal duplications with co-existing monosomy of another chromosome region cause severe phenotypes whereas patients with pure interstitial duplications seem to be only mildly affected [6–8]. Unfortunately the breakpoints of the majority of published cases were assessed with karyotyping or FISH, which hampers finding critical regions for ID and other features [9] so the genotype-phenotype studies in cases characterized by microarray are of great value [10].

We report on two families with an interstitial imbalance involving region 6q21q23: a familial duplication of 6q22.1q23.2 and one of 6q21q22.33. This is the first report where the gene content and breakpoints of a microscopically visible interstitial 6q duplication was characterized with microarray. In these families, some individuals carrying the imbalance did not show any structural anomalies or ID associated with trisomy 6q as described before, suggesting incomplete penetrance.

## Case presentation

### Family 1

A 23 year-old pregnant woman was referred to the department of Clinical Genetics due to isolated ID and a familial interstitial duplication on chromosome 6 characterized by karyotyping in the past. This duplication was discovered in 1983 in a family member that was known to suffer from ID, developmental delay (DD) and seizures. The observed ID in this family varied from very mild (low degrees and learning problems) to more severe (accompanied by DD and seizures).

The partner of the pregnant patient was known to have ID as well. He was an adopted child. His biological brother was said to have ID as well. Neither further family history, nor parental samples were available. Because of the pregnancy, our patient and her partner were investigated with genomic SNP array (Illumina HumanCytoSNP-12) to study their chromosomal constitution.

The pregnant patient carried an interstitial ~16.1 Mb 6q duplication and the array testing additionally revealed a susceptibility locus (SL) for neurodevelopmental disorders: a ~580 kb 16p11.2 microduplication (arr[hg19] 6q22.1q23.2(116,411,005-132,541,408)x3,16p11.2(29,595,483-30,174,926)x3). The partner of the pregnant patient was shown to carry another SL for neurodevelopmental disorders, a ~806 kb 16p11.2 microdeletion (arr[hg19] 16p11.2(28,475,873-29,282,147)x1).

**Fig. 1:**
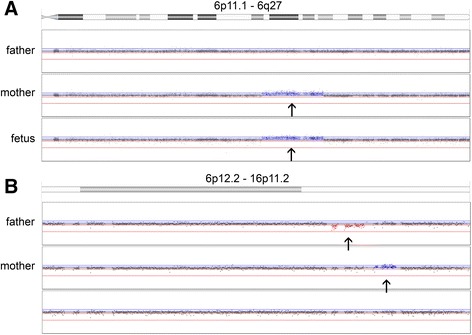
Family 1: Log Ratio plots of Illumina CytoSNP-12: **a** – partial chromosome 6q plot showing the 6q duplication in family 1: the mother and the fetus carry a 6q22q23 duplication. The arrows indicate the duplicated region. **b** - partial chromosome 16p plot showing the imbalances in 16p11: the father carries 16p11 microdeletion, the mother 16p11 microduplication, whereas the fetal array profile is normal for both 16p regions. The arrows indicate the imbalances in parental samples

Because both partners had abnormal array results, prenatal diagnosis was advised. Amniocentesis was performed at 15 5/7 weeks of gestation and diagnostic array testing on uncultured amniotic fluid cells showed only the interstitial 6q duplication (Fig. 1). In order to enable clinical interpretation of this result, 4 other family members of the pregnant woman, two healthy and two affected (one mild and the other severe (the one that was karyotyped in 1983, see above)), were tested with SNP array. Two healthy relatives turned out to carry only the 6q duplication, whereas the two affected relatives carried both the 6q duplication and the 16p11.2 microduplication.

As the fetus inherited only the 6q duplication, in the absence of SL, the prognosis of the fetal phenotype may be favorable. The second trimester fetal ultrasound investigations showed no structural anomalies. A healthy baby boy without any dysmorphic features was born and there were no developmental abnormalities noted so far (follow up at the age of 1 year).

### Family 2

**Fig. 2:**
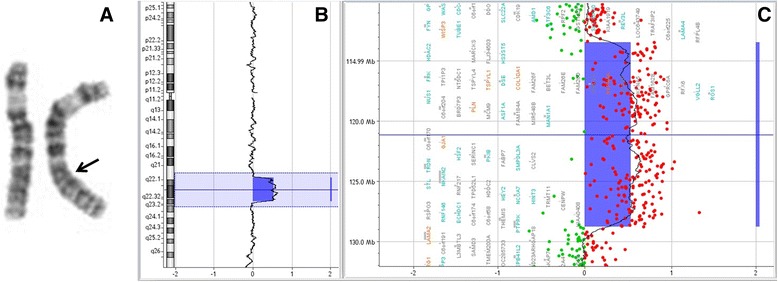
Family 2: **a** -Karyotype in standard resolution for chromosome 6, showing the interstitial duplication (arrow). **b** -array results (Oligo aCGH Agilent 60 K) for chromosome 6 in family 2 showing duplication of 6q present in family 2. **c**- The duplicated region and its gene content

A 34 year-old pregnant woman was referred to a clinical geneticist after suspicion of fetal esophageal atresia at third trimester ultrasound investigations, based on polyhydramnios and lack of visualization of the stomach. This was the first pregnancy of non-consanguineous parents. Fetal karyotype (amniotic fluid) revealed an interstitial duplication on chromosome 6. SNP-array (Affymetrix CytoScan HD) was performed, showing a 15.3 Mb duplication (arr[hg19]6q21q22.33(113,465,762-128,774,807)x3) (Fig. 2). Given the combination of the duplication along with fetal structural abnormalities, the parents opted for termination of pregnancy at 33 weeks gestational age. The male fetus had normal biometry: weight 2593 g, height 49 cm, occipitofrontal circumference (OFC) 29 cm. Dysmorphic facial features were noted: short nose with anteverted nares and long eyelashes. Fetal autopsy confirmed the esophageal atresia and revealed additional structural abnormalities: an abnormal pulmonary lobulation (2 lobes on right lung and 1 lobe on left lung), a Meckel’s diverticulum, bilateral hydronephrosis, pancreatic ectopia, and neuronal heterotopia of the cerebellum located on the dentate nuclei.

Familial studies revealed that the mother was carrier of the same interstitial duplication on chromosome 6 (karyotyping and Oligo aCGH Agilent 60 K), while her parents had normal karyotypes. No additional copy number variation was found in both the mother and the fetus. The mother had previously experienced learning difficulties, dyslexia and was known with a scoliosis. Cardiac ultrasound showed no abnormalities. No cerebral or abdominal imaging was performed. She works as a nurse assistant in a hospital.

## Discussion

In many laboratories over the world, genomic array testing is now a first-tier cytogenetic test. The interpretation of array results is mainly based on comparisons to CNVs in control and patient populations [11]. Comparison to previously reported cases with microscopically visible chromosome anomalies, however, is problematic. The breakpoints of the majority of published cases were assessed with karyotyping or FISH, which hampers the genotype-phenotype correlation studies as these breakpoints often are not accurate enough to compare them with array results [9]. In the karyotyping era a *de novo* unbalanced euchromatic chromosome aberration used to be almost always interpreted as pathogenic, however rare inherited variants were assumed to be benign and interpreted as unbalanced euchromatic abnormalities without phenotypic consequences (if inherited from a normal parent) [12–17]. At that time susceptibility CNVs (variants of variable expressivity and phenotypic heterogeneity) [18, 19], which expression seems to depend on other (genetic and/or environmental) factors, were less commonly known.

### Phenotypes of 6q21q23 duplication carriers

**Fig. 3:**
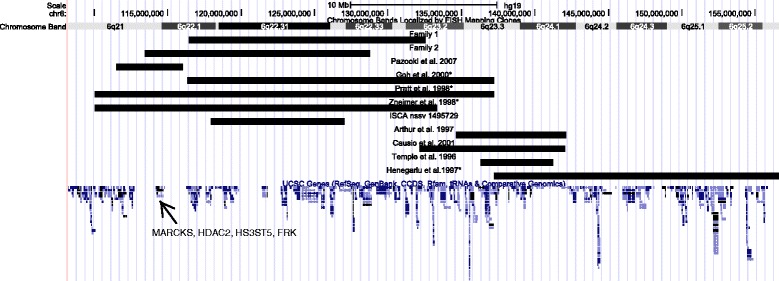
Breakpoints comparison (in GRCh37/hg19) in cases with dup 6q21q23 where molecular testing was done (minimal breakpoints based on FISH/YAC or DNA markers or given by the authors of original papers, if appropriate lifted from other genome builds)

The literature searched showed only 12 cases of interstitial duplications in chromosome region 6q21-6q23 (Table 1, Fig. 3) [7, 20–26]. The phenotype show variability and as shown in Fig. 3 not all duplication cases overlap with the families presented here. Congenital malformations seem to be infrequent, with only few patients reported to have variable cardiac anomalies (right ventricular hypertrophy, cardiomegaly, ventricular septal defect, pulmonary stenosis) [24, 25], and one suspicion of a corpus callosum agenesis [24]. The 6q duplications in the current families seem to overlap with the ISCA case and patients published by Goh et al., Pratt et al. and Zneimer et al. [20, 24, 25], although the breakpoints based on karyotyping could be highly imprecise. All these patients presented with DD/mild ID and have common craniofacial features with constant hypertelorism and frequent mid-face hypoplasia with anteverted nares [20, 24, 25]. Further, the two cases reported by Pazooki et al. that only partially overlap with the duplication in family 2 also presented DD and similar dysmorphic features (hypertelorism and anteverted nares), but no congenital malformation.

**Table 1:** Cases of interstitial duplications in chromosome region 6q21-6q23

	Cytogenetic results	Molecular testing	Minimal duplication region according to molecular tests	Molecular breakpoints^a^in GRCh37/hg19	Phenotype of the proband
Temple et al. 1996	46,XX, der(2) ins(2;6)(2pter-2p22.2::6q?22.32- ?6q?23.1::2p22.2–2qter)pat^b^	Markers analysis	dup(6)(q23.3q24.1) (between markers D6S292 and D6S308)	chr6: 136315214-141256931	small at birth, neonatal diabetes, microcephaly, myoclonic seizures, no DD (the insertion is resulting in partial trisomy: dup(6)(6q22.33–q23.3))
Arthur et al. 1997	46,XX, inv dup(6)(q22q23)pat	Markers analysis	invdup(6) (q23.2q24.1) (between markers D6S270 and D6S310	chr6: 134654821- 142102601	transient neonatal diabetes mellitus, minor dysmorphic features
Henegariu et al.1997	46,XX,dir dup(6) (q23.3q25.3)^c^	FISH with painting probe for chr6	ukn	ukn	dolichocephaly, dyspmorphic features, long fingers, bilateral single palmar creases, contractures at the wrists, long fingers, dorsiflexion of the first toes, mild syndactyly of toes 2–3, bradycardia, mild tricuspid insufficiency, a small patent ductus arteriosus, and small atrial septal defect, moderate cardiomegaly, short neck but not webbed, umbilical hernia, and mild generalized hypertonia
Pratt et al. 1998 (2 cases)	Case 1: 46,XY,dup(6)(q21q23.3)dn Case 2: 46,XX,dup(6)(q21.15q23.3)dn	FISH with painting probe for chr6	ukn	ukn	Case 1: dysmorphic features, bilateral clubfoot, ulnar deviation of hands, decreased muscle tone, decreased subcutaneous fat, brain anomalies, a thickened tricuspid valve, bilateral transverse palmar creases, ulnar deviation of the hands, club feet, and small hands and feet Case 2: DD/ID (IQ 63), attention deficit disorder with hyperactivity, and coördination difficulties. Dysmorphic features, ventricular septal defect and pulmonic valvular stenosis, clinodactyly of the 5th finger, and thickened nails of the 5th toes, increased reflexes and ankle clonus.
Zneimer et al. 1998	46,XX,dup(6)(q21q23)dn	FISH with painting probe for chr6	ukn Possibly dup(6)(q22q24) ivm neonatal diabetes mellitus^d^	ukn	born with umbilical hernia and transient insulin dependent diabetes until 7 months, delayed development and speech (receives speech therapy), dysmorphic features, cardiomegaly of unknown etiology
Goh et al. 2000	der(7)ins(7;6)(q21.11;q22.1q23.3)mat resulting in partial trisomy of 6q22.1q23.3	N.D.	ukn	ukn	Case 1: obesity, brachycephaly, dysmorphic features, bilateral clinodactyly, DD, ID with IQ of 50.8, obstructive sleep apnoea Case 2: (older sister of case 1) brachycephaly, dysmorphic features, bilateral clinodactyly, mild DD, no formal IQ test, but left school without academic credits.
Causio et al. 2001	46, XY, dup(6)(q23q23)dn	FISH with YAC probes	dup(6)(q23.2q24.1) according to minimal duplication breakpoints using YAC probes (970A12-949D3)	chr6: 132138813-142077202	plagiocephaly, DD, ID, hiatus hernia (corrected surgically), acrocephaly, dysmorphic features, short hands with clinodactyly of fifth finger, joint contractures. Nuclear cataract in OD and rotating nystagmus in OO, bicuspid aortic valve with moderate failure, psychotic behavior and neurological tics.
Pazooki et al. 2007 (mother and child)	46,XXdup(6)(q21q22.1)mat	FISH with BAC probes	dup(6)(q21q22.1) according to minimal breakpoints using BAC probes (RP11-428 F8 and RP1-64I15)	chr6: 111498415- 116048892	Child: DD, stridor, nutritional problems, obesity, intention tremor, dysmorphic features (anteverted nares, depressed nasal bridge, hypertelorism, short fingers).
					Mother: cognitive difficulties, obesity, intermittent tremor.
ISCA nssv 1495729	arr[hg19] 6q22.1q22.32(117,928,310-127,021,125)x3dn	Oligo aCGH	dup(6) (q22.1q22.32)	chr6: 117928310-127021125	DD and/or other significant developmental or morphological phenotypes
Current family 1	arr[hg19] 6q22.1q23.2(116,411,005-132,541,408)x3mat	Illumina Human CytoSNP-12	dup(6)(q22.1q23.2)	chr6: 116411005-132541408	-normal phenotype in 6q duplication carriers, -variable phenotype (from learning problem to developmental delay and intellectual disability) in carriers of both 6q duplication and 16p11.2 microduplication.
Current family 2	arr[hg19] 6q21q22.33(113,464,340-128,801,386)x3mat	Affymetrix SNP CytoScan HD	dup(6)(q21q22.33)	chr6: 113464340-128801386	Fetus: dysmorphic features (anteverted nares, short nose, long eyelashes), esophageal atresia, pulmonary lobulation anomaly, meckel diverticulum, bilateral hydronephrosis, pancreatic ectopia, neuronal heterotopia of the cerebellum (dentate nucleus). Termination of pregnancy at 33WG.
	arr[hg19] 6q21q22.33(113,465,762-128,774,807)x3dn	Oligo aCGH Agilent 60 K	dup(6)(q21q22.33)	chr6: 113465762-128774807	Mother: learning difficulties, dyslexia, scoliosis.

*N.D*. not done, *dn* de novo, *mat* maternal, *pat* paternal, *DD* developmental delay, *ID* intellectual disability, *ukn* unknown

^a^The molecular breakpoint were either given by the authors or translated from molecular markers/FISH probes and show minimal region of imbalance

^b^ Father and grandfather had duplicated 6q, but no abnormal features or neonatal diabetes

^c^ Not maternal, father not tested, if including band 6q24 probably *de novo* on maternal chromosome as neonatal diabetes mellitus was not stated

^d^ The work of Docherty and colleagues proved that neonatal diabetes mellitus is associated with 6q24 (Docherty, Poole et al. 2010), it may be assumed that the actual duplicated region does not contain bands q21q23, but more distal region on the long arm of chromosome 6 including 6q22q24

### Possible candidate genes for DD/ID in 6q21q23

Although the duplicated regions are large, we conducted a gene content study to search for candidate genes that may explain the ID/learning problems in presented families. Gene lists were generated using Nexus BioDiscovery Copy Number 7.5 and the data were uploaded and analyzed with QIAGEN’s Ingenuity® Pathway Analysis (IPA®, QIAGEN Redwood City, www.ingenuity.com). A list of genes and proteins related to ID was generated using the Knowledge Base of IPA. In case of the 6q duplication in family 1, we did not find any candidate gene that may explain the ID when duplicated, which may support our hypothesis that the 6q duplication seems to be benign if present alone. However, the proximal region that was duplicated in family 2, but not in family 1 seems to have an interesting gene content. It contains four OMIM genes: *MARCKS, HDAC2, HS3ST5* and *FRK,* all involved in brain function and development [27–30]*.* Interestingly it was shown that *MARCKS* expression is significantly elevated (45 %) in the hippocampus of mice, which exhibit impaired hippocampus-dependent learning [27] and neuron-specific over-expression of *HDAC2* decreased dendritic spine density, synapse number, synaptic plasticity and memory formation [31]. Since all four genes are involved in neural development, one could speculate that if their expression is dosage dependent, their duplication may play a role in neurological phenotypes like ID, DD and brain malformations. To our knowledge, so far, there are no cases of whole gene duplications in *MARCKS, HDAC2* and *HS3ST5* in control populations (neither in Decipher Population CNVs, Toronto Database of Genomic Variants, nor our in house controls), and there is only one family published by Pazooki et al. carrying a smaller duplication, but overlapping also with *MARCKS, HDAC2, HS3ST5* (Fig. 3) [26]. Unfortunately these data are not enough evidence to claim that duplication of *MARCKS, HDAC2, HS3ST5* causes learning problems or ID/DD. The duplication of these genes remains a variation of unknown clinical significance, however it cannot be excluded that it may contribute to learning problems when also other genes involved in neurological phenotypes are deregulated.

### The second hit hypothesis

The variability in phenotypes in patients shown in Table 1 could be explained by different chromosome breakpoints, however the duplications in the current families show variability within a family as well. This cannot be explained by different gene content, but it may be a consequence of the presence of additional anomalies in affected individuals.

The two-hit model has been introduced to explain the phenotypic variability in genetic disorders [18, 19]. The presence of the additional hit may modify phenotype, either acting in an additive manner (independently adding new features), or by participation in the same or similar biochemical pathway causing more severe phenotypes [32]. And indeed in family 1, the 6q22.1q23.2 duplication seems to be an euchromatic variant without phenotypic consequences, as only the individuals carrying the 6q duplication and the additional susceptibility CNV showed abnormal phenotypes. Family 1 shows very high penetrance of the susceptibility locus, when both chromosome aberrations are present. Although this observation can be reassuring for the fetus, we found prediction of the fetal prognosis still problematic since the father of the fetus carried a susceptibility locus for neurodevelopmental disorder, but the paternal “additional hit” remained unknown. The fetus did not inherit the paternal SL, nevertheless the paternal “additional hit” may have been inherited and may influence the intellectual development of the child.

In family 2, the interstitial 6q duplication seems to show highly variable expression in the mother and fetus. The fetus had dysmorphic features similar to reported cases in the literature and multiple congenital malformations that were not described before. The breakpoints and gene content were identical in the mother, who only had learning difficulties in childhood. There are no healthy carriers of this duplication in this family and the *de novo* occurrence of this chromosomal duplication in the mother of the fetus may be an argument for its causality for the phenotype. The “second hit” has not been identified by microarray in this family and remains unresolved. Genetic counseling for a future pregnancy remains problematic: it is doubtful whether it is reasonable to offer an invasive prenatal test, if the “additional hit” is still unknown and if no fetal ultrasound abnormalities are present. Given these limitations, the couple has opted for preimplantation diagnosis.

## Conclusions

Finding a rare euchromatic variant or a susceptibility locus is challenging in both postnatal and prenatal settings. Our study shows that the 6q22.1q23.2 duplication may represent a private variant that is benign when present alone, but that may act as the “additional/second hit” in SL carriers. We suggest that also large benign CNVs can serve as “an additional hit” and therefore our study supports the opinion of Thomas Liehr that benign variants cannot be fully neglected in genetic analysis [17]. The presented family 1 supports “the multi-hit hypothesis” showing that the penetrance of such a SL in families known to carry an “additional hit” can be much higher than predicted [33], which may explain the conflicting conclusions on the association with an abnormal phenotype in the papers on SL [34–36]. Finding the “additional hit” within the family is therefore very important for genetic counseling and the assessment of the SL penetrance within the particular family.

## Abbreviations

CNV, copy number variation; DD, developmental delay; FISH, fluorescent in situ hybridization; ID, intellectual disability; OFC, occipitofrontal circumference; SL, susceptibility locus
